# Flexibility and structural conservation in a c-KIT G-quadruplex

**DOI:** 10.1093/nar/gku1282

**Published:** 2014-12-01

**Authors:** Dengguo Wei, Jarmila Husby, Stephen Neidle

**Affiliations:** UCL School of Pharmacy, University College London, London WC1N 1AX, UK

## Abstract

A quadruplex sequence from the promoter region of the c-KIT gene forms a stable quadruplex, as characterized by crystallographic and NMR methods. Two new crystal structures are reported here, together with molecular dynamics simulation studies on these quadruplex crystal structures and an NMR structure. The new crystal structures, each in a distinct space group and lattice packing arrangement, together with the existing structures, demonstrate that the c-KIT quadruplex fold does not change with differing environments, suggesting that quadruplex topological dynamism is not a general phenomenon. The single and dinucleotide loops in these structures show a high degree of conformational flexibility within the three crystal forms and the NMR ensemble, with no evidence of clustering to particular conformers. This is in accord with the findings of high loop flexibility from the molecular dynamics studies. It is suggested that intramolecular quadruplexes can be grouped into two broad classes (i) those with at least one single-nucleotide loop, often showing singular topologies even though loops are highly flexible, and (ii) with all loops comprising at least two nucleotides, leading to topological dynamism. The loops can have more stable and less dynamic base-stacked secondary structures.

## INTRODUCTION

Quadruplex DNA and RNA structures are formed from repeats of guanine tracts that are interspersed by mixed sequences (‘loops’). These serve to connect together the basic building-blocks of these four-stranded arrangements, the G-quartet (1,2). Sequences encoding for quadruplex nucleic acids have been found in a variety of locations in the human (3,4) and other genomes (5), principally in eukaryotic telomeres (6,7), in promoter regions (8–10), in untranslated regions especially 5′ and 3′-UTRs (11,12) and in a number of breakpoint regions (13–16). Their biological functions remain to be fully elucidated, although the demonstration of the existence of quadruplexes in cancer cells (17,18) and of enhanced levels in human cancer tissues (19) is strongly supportive of the concept that the presence of quadruplexes may be deleterious when they cannot be fully unwound, as in cancer cells having for example deficiencies in the FANCJ helicase.

The concept that sequences within a promoter comprising repeats of short G-tracts could form higher-order quadruplex structures was first suggested for the c-MYC gene (9,20). Such sequences have subsequently found to be widely prevalent in the human genome, with a particular over-representation in genes involved in proliferation, notably in oncogenes such as k-RAS (21), b-RAF (22), SRC (23), BCL-2 (24,25) and c-KIT (26–32). Promoter quadruplexes can be stabilized by the binding of appropriate small molecules and it has been suggested that this can be an effective approach to transcriptional inhibition at the single-gene level (9,10). A large number of studies have reported down-regulation of expression of one or other these genes in cell-based systems when quadruplex-binding small molecules have been used (see (10,^33^,34) for recent reviews). Correlations have been suggested in a number of these studies between *in vitro* small molecule quadruplex affinity to a particular promoter quadruplex and expression changes in that particular gene, although this is not always the case (35). Even though there is no definitive evidence to date of cause and effect in cells for a particular promoter quadruplex gene target, the attractiveness of the concept has led to its current emphasis as a novel drug discovery strategy.

The c-KIT gene codes for the KIT tyrosine kinase gene product, which is implicated in a number of human cancers, notably gastro-intestinal cancer (GIST), where dis-regulation of c-KIT expression is the primary causative event of this disease (36–38). Two quadruplexes have been identified in the promoter of this gene, one positioned between −87 and −109 bp (termed here c-KIT G4) (26) and the other −140 and −160 bp (termed here c-KIT1 G4) (27), upstream of the transcription initiation site. A number of studies with low molecular-weight compounds have shown correlations between their c-KIT/c-KIT1 G4 binding and inhibition of c-KIT expression (39–44) and it has been suggested that quadruplex targeting may be a novel approach to the therapy of GIST.

**Figure 1.**
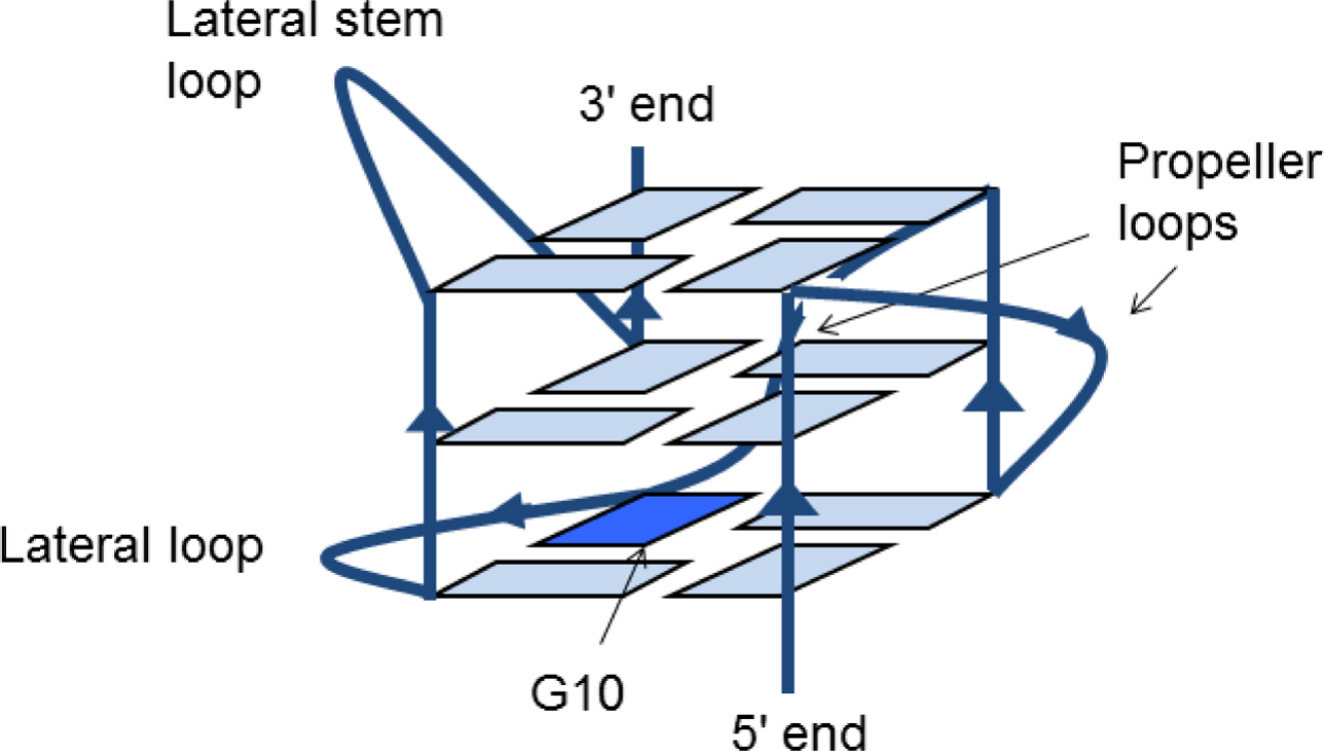
Schematic representation of the c-KIT quadruplex topology.

The topology and molecular structures of the c-KIT (28,31) and c-KIT1 (29,30) G4s have been characterized by nuclear magnetic resonance (NMR) studies and both show unique features compared to other known quadruplexes. In addition a crystal structure of the c-KIT G4 has been determined (32), with topology identical to that reported in the NMR study (on the identical sequence), albeit with differences in groove dimensions. The quadruplex has a parallel fold with two single-nucleotide propeller (double chain-reversal) loops and a long five-nucleotide lateral stem loop; unusually one non-G-tract guanine participates in the core of stacked G-quartets (Figure 1). The equivalence of solution and crystal structures suggests that the dynamics of the c-KIT quadruplex are limited and that the observed topology represents the global minimum structure. We report here on two further c-KIT crystal structures that have also been determined on the identical sequence, but crystallize in distinct space groups (*P*3_1_21 at 1.82-Å resolution and *H*3 at 2.73 Å, compared to space group *P*2_1_2_1_2 for the previously reported brominated form, which was determined at 1.62-Å resolution). These new crystal structures also show the unique c-KIT topology, providing added credence to this hypothesis. We further report on a series of molecular dynamics (MD) simulations, on a total of five distinct c-KIT quadruplex starting-points from the two high-resolution crystal structures and the NMR structure. This has enabled the flexibility of the c-KIT quadruplex to be examined in detail, and implications for the structure-based design of specific small molecules to be addressed.

## MATERIALS AND METHODS

### Crystallography

The c-KIT DNA quadruplex sequence d(AG_3_AG_3_CGCTG_3_AGGAG_3_) was synthesized and purified as described previously (32). A 2-mM DNA solution containing 20-mM potassium cacodylate buffer at pH 6.5 and 50-mM KCl was heated to 358 K before annealing by slow cooling to room temperature. Crystals were grown by the hanging-drop vapor diffusion method. 1 μl of a premixed drop solution containing 10% MPD, 10-mM MgCl_2_ 50-mM KCl and 50-mM sodium cacodylate at pH 6.5 was added to 1 μl of c-KIT DNA solution at a concentration of 2 mM. The drop solution was equilibrated against the well solution with 50% MPD, 20-mM MgCl_2_ and 100-mM NaCl at 283 K. Hexagonal and rod-like crystals appeared after 2 weeks. The rod-like crystals were assigned the alternative hexagonal *H*3 setting for the rhombohedral space group *R*3 and the hexagonal crystals were assigned the trigonal space group *P*3_1_21. Data sets were collected at 105 K on a single flash-frozen crystal of each type at the Diamond synchrotron facility (UK).

Molecular replacement methods were used in solving these two crystal structures using the G-quartets extracted from the earlier ^Br^U c-KIT DNA crystal structure (PDB code 3QXR). Other parts of the quadruplex structures were progressively built on the basis of 2F_o_-F_c_ and F_o_-F_c_ difference electron density maps. This was followed by the location of K^+^ ions in the central ion channels. A total of 162 water molecules were identified in the 1.82-Å hexagonal crystal structure, and three water molecules were located in the 2.73-Å trigonal crystal structure. Final *R* and *R*_free_ values are listed in Table 1. Coordinates and structure factors are available from the PDB as entries 4WO2 and 4WO3, respectively. Diffraction data were processed by the SCALE program in the CCP4 package (45). Programs COOT (46), PHASER (47) and REFMAC5 (48) were used in the processes of structure building, model fit and refinement, respectively. Programs CHIMERA (49) and PYMOL (50) were used for visualization and analysis. Table 1 details the crystallographic data.

**Table 1.** Crystallographic data collection and refinement statistics

Sequence	d(AGGGAGGGCGCTGGGAGGAGGG)		
	^Br^U c-KIT	c-KIT	c-KIT
**Data collection**			
Space group	*P*2_1_2_1_2	*P*3_1_21	*H*3
Cell dimensions			
a, b, c (Å)	37.57,51.35, 58.34	31.44,31.44,186.81	94.85,94.85,30.36
Wavelength (Å)	0.9763	0.9173	0.9686
Resolution (Å)	38.55–1.62	31.13–1.82	28.48–2.73
*R*_merge_	0.057	0.083	0.115
*I*/*σ*	27.4	14.5	5.0
Completeness (%)	93.2	99.1	94.4
Redundancy	3.6	6.9	2.6
Total no. of reflections	50085	72204	6630
No. of unique reflections	14007	10436	2561

**Refinement**			
Resolution (Å)	26.9 – 1.62	10.28–1.82	28.48 – 2.73
No. of reflections	13279	9807	2439
*R*_work_/*R*_free_	0.176/0.221	0.210/0.223	0.183/0.246
No. of ions	11.5	6	5
No. of water molecules	210.5	162	3
Overall B factor (Å^2^)	20.07	27.17	41.24
RMS deviations in			
Bond length (Å)	0.005	0.006	0.008
Bond angle (º)	1.0	0.694	1.3
PDB ID	3QXR	4WO2	4WO3

The data on the ^Br^U c-KIT structure have been previously reported (32): these are shown here for comparison purposes.

### MD simulations

Coordinates for the two higher-resolution crystal structures and the NMR structure of the c-KIT quadruplex DNA were obtained from the Protein Data Bank (PDB). Those were:

- the crystal structure of ^Br^U c-KIT G4 (PDB 3QXR) at 1.62-Å resolution (32);
- the crystal structure of the native c-KIT G4 at 1.82-Å resolution (PDB 4WO2);
- the NMR structure of monomeric c-KIT G4 (PDB 2O3M) (28).

All four crystallographically independent quadruplexes were used in the simulations since both of these X-ray structures comprising two intramolecular quadruplexes per asymmetric unit (chains A and B), together with model 1 from the NMR structure. These intramolecular 22-base quadruplexes all have the identical native sequence, 5′-d(AG_3_AG_3_CGCTG_3_AG_2_AG_3_)-3′, which has not been modified from that occurring in the c-KIT gene promoter sequence. Nucleotide numbering in this paper is sequential from the 5′-adenosine.

The sole exception to this is the sequence in the brominated X-ray structure (32), which differs at the 12th position, where 5-brominated uridine (^Br^U) replaced a thymine (T). The ^Br^U residue was modified back to T for the simulations using the Accelrys Discovery Studio package (www.accelrys.com; v.3.5), to match the original native sequence. Overall, five individual c-KIT 22-mer quadruplex units were used as starting points for the MD studies, i.e.

#1 chain A and #2 chain B, both derived from the ^Br^U c-KIT crystal structure,
#3 chain A and #4 chain B, derived from the high-resolution native c-KIT crystal structure reported here,
#5 model 1 from the native c-KIT NMR structure.

Since the individual conformers available in the NMR ensemble have an average deviation in their structural alignment of <0.5 Å, it was considered that any one of them would be equally suitable to be used as a starting point for the MD study. The NMR structure was used for comparison purposes—to explore the dynamic behavior of c-KIT structures of identical sequence, but determined by two different techniques (X-ray and NMR).

Consecutive K^+^ ions vertically aligned within the central core of the quadruplexes and mid-way between each G-quartet were retained at their respective crystallographic positions, as well as the structural K^+^ ions present in the loops of individual 1, 2 and 3 quadruplexes. Two additional Mg^2+^ ions observed in the loops of quadruplex 1 were also retained for the simulations. In the NMR system, the missing structural K^+^ ions were manually added into the central core of the G4, within their respective positions as reported in the X-ray structures. The explicit water molecules, present within the crystallographic structures, were retained for the simulation setups, even though they only represent a small fraction of the total numbers of water molecules in these structures.

All of the MD simulations were all-atom ones, performed with the GROMACS v.4.6 program (51), employing the AMBER *parmbsc0* force field (52) ported into GROMACS. The TIP3P water model was used and for the ions, AMBER parm99sb parameters were applied, both for the structural ions and the counter-ions. The simulation protocols, reported elsewhere (53,54), were consistent for all five c-KIT quadruplex systems. The production step of each of the five MD simulations was carried out in triplicate for 250 ns in order to improve sampling and statistical reliability, resulting in 750 ns of simulation data per quadruplex for subsequent analysis. Altogether, 3750 ns of MD trajectories were generated and analyzed by a robust clustering algorithm that automatically provides the desired number of clusters, in order to identify the most prevalent conformations sampled throughout the simulation time at 300 K. All the simulations were performed on an in-house AMD CPU-based Linux cluster (IBM Blade Center H; 16 CPUs per simulation) at the Italian Institute of Technology (Genoa, Italy). A robust clustering algorithm (55), adapted to cope with MD data (56), was employed here (see the Supplementary data for further information). The advantage of using this particular clustering algorithm is that multiple non-consecutive trajectories of an individual system can be clustered together, while it ensures that only those transitions between individual clusters are counted/reported if the frames belong to the same/corresponding trajectory, and also ensures that no ‘artificial’ (or unreal) transitions between clusters are formed.

Clustering of the MD trajectories (3 × 250 ns) was carried out for each of the five systems, employing a total of 150 000 frames, each having a 5-ps time step. The maximum number of clusters was set up to 10 (this was found to be an optimal number, based on preliminary clustering analysis where various numbers of resulting clusters were identified—ranging from 5 to more than 20).

## RESULTS

### Crystal structures

**Figure 2.**
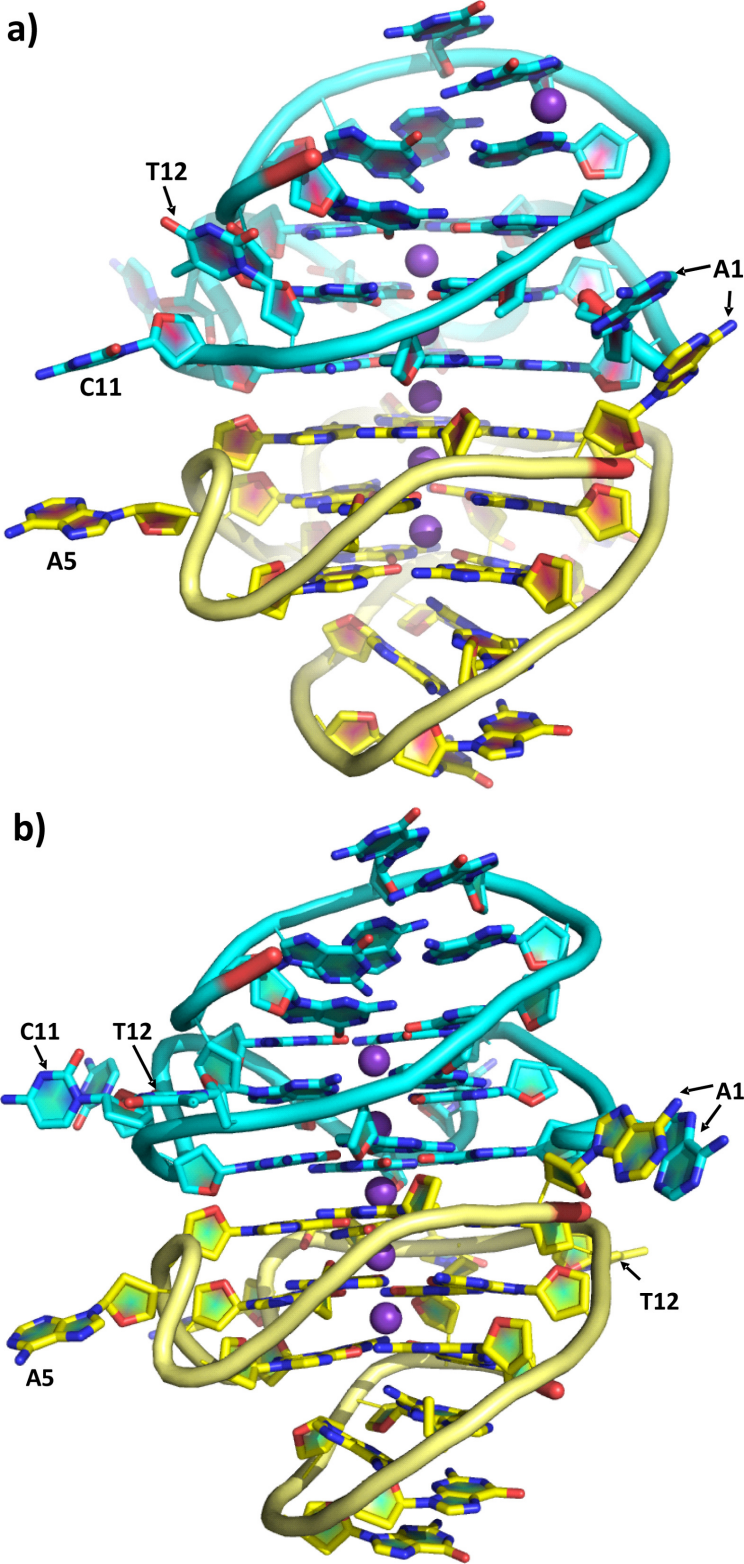
Cartoon representation of the crystallographic asymmetric unit of the two c-KIT quadruplex crystal structures. The two chains in each instance are colored cyan and yellow and the potassium ions are shown as purple spheres. (**a**) The 1.82-Å crystal structure in space group *P*3_1_21; (**b**) the 2.73-Å crystal structure in space group *H*3.

The c-KIT quadruplex in both the 1.82-Å and the 2.73-Å structures (Table 1) forms head-to-head dimers in the crystals, closely analogous to that previously observed in the ^Br^U form. Each monomeric intramolecular quadruplex in a dimer has been designated as chain A or B. Thus the two new crystal structures comprise four crystallographically independent c-KIT quadruplexes. The quadruplex topology in each instance is identical to that previously reported for c-KIT in the crystal and in NMR solution. In each instance the 22-nucleotide DNA sequence folds into a parallel four-stranded G-quadruplex, and the isolated non-G-tract guanine (G10) is embedded in the G-quartet core. Two single-residue linkers (A5 and C9) form two propeller (double-chain reversal) loops that bridge three G-quartet layers. C11 and T12 form the third loop, which connects two G-quartet corners (G10 and G13). The stem-loop formed by the five-residue sequence A16–G17–G18–A19–G20 allows the terminal G21–G22 to be inserted back to the G-quartet core. The fourth loop, the long lateral stem loop retains its secondary particular features of two A:G hydrogen bonds. Potassium ions were located in the central channel of both crystal structures (Figure 2). No additional potassium or magnesium ions were observed in the grooves or loops of these quadruplexes. This contrasts with their location in the higher-resolution ^Br^U form (strong electron density was observed in the 1.82-Å crystal structure at the boundary between two stacked asymmetric units, which is consistent with a potassium ion rather than a water molecule).

**Figure 3.**
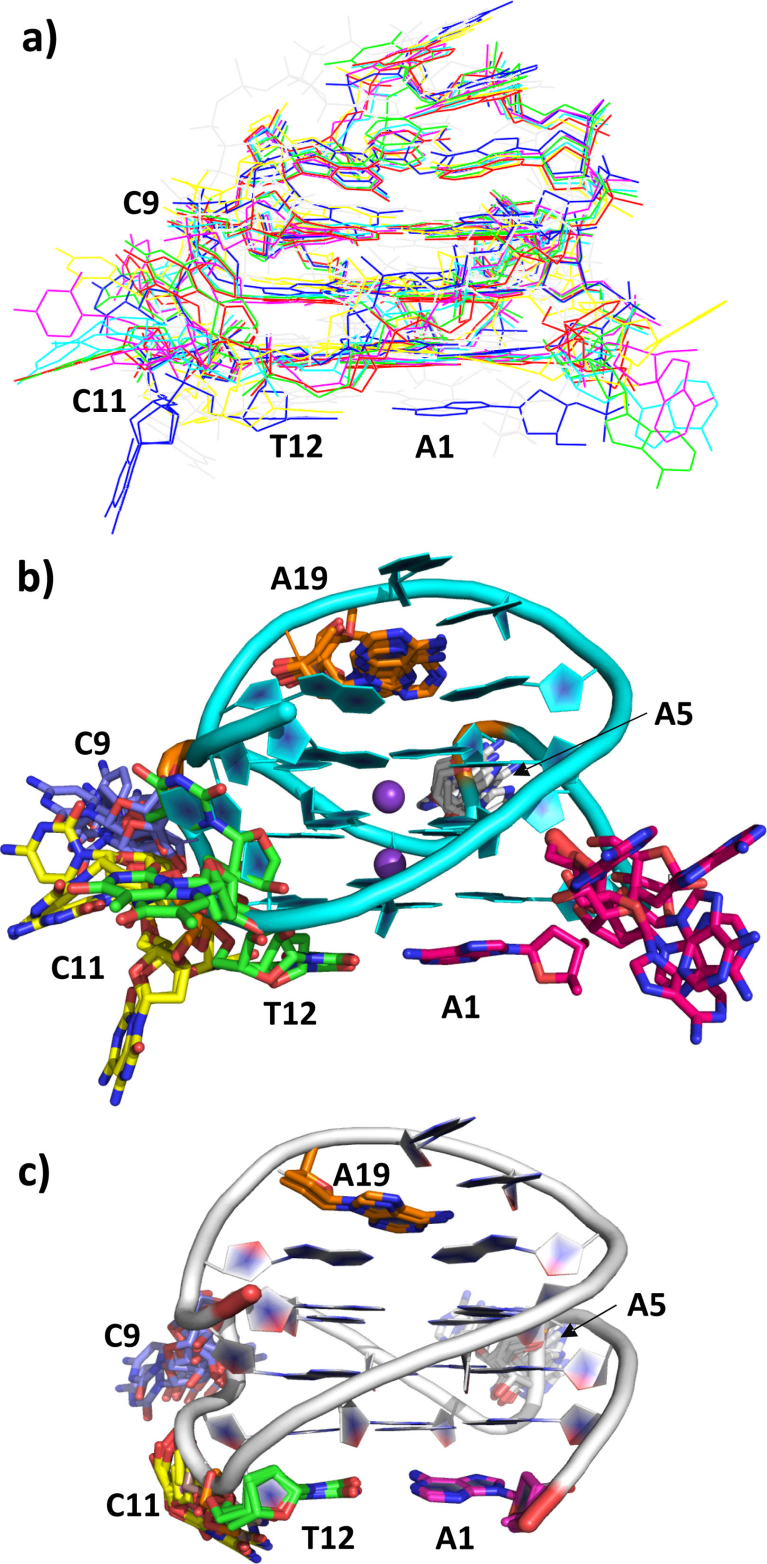
(**a**) Composite plot showing a superposition of the two c-KIT crystal structures, the previous ^Br^U c-KIT crystal structure and the c-KIT NMR structure used in the MD analysis. (**b**) Composite plot showing a superposition of the three c-KIT crystal structures, highlighting the nucleotides with variable conformations. (**c**) composite plot showing a superposition of the ensemble of c-KIT NMR structures. In (b) and (c) all bases are shown but with only a single backbone.

**Figure 4.**
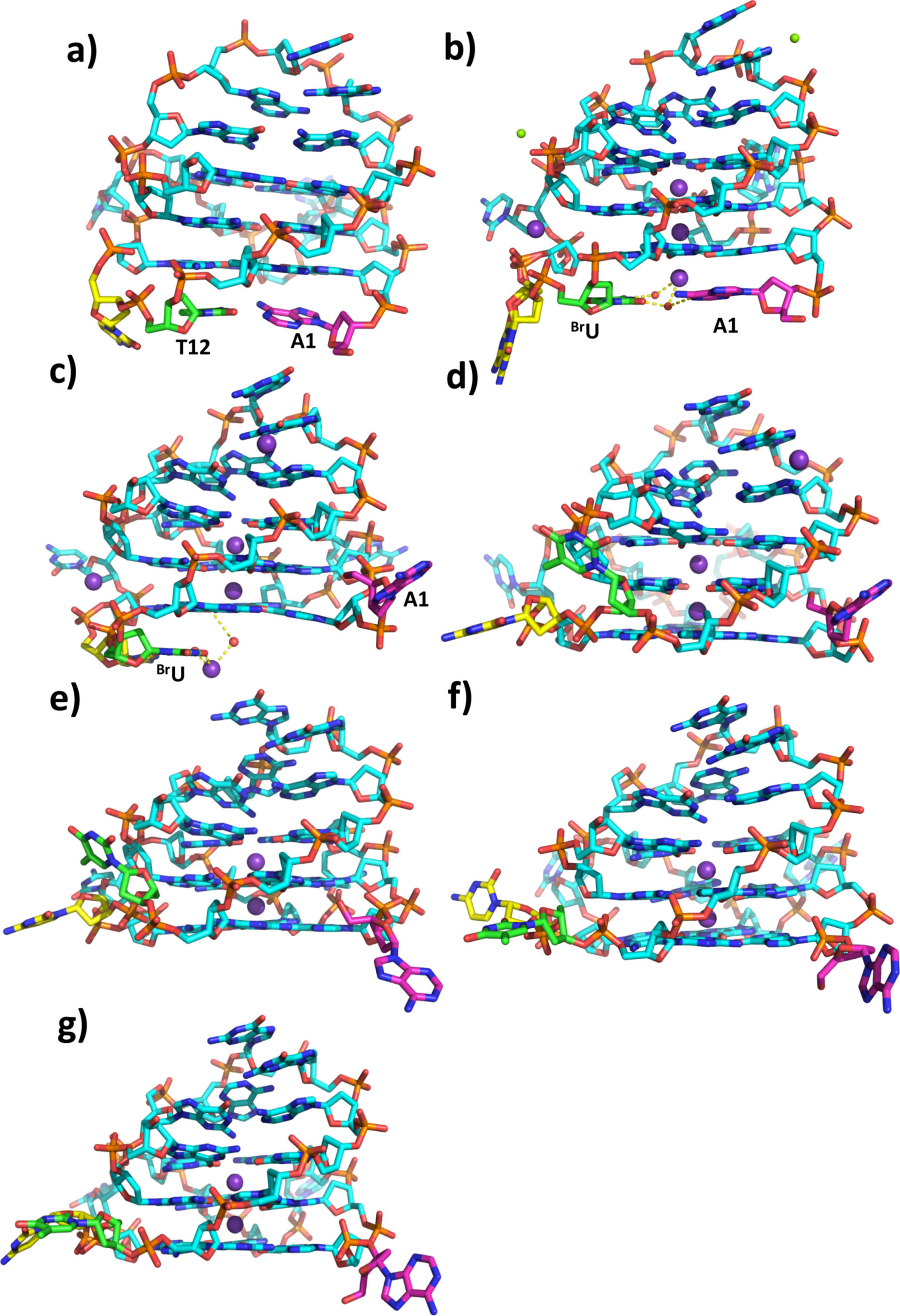
The flexibility of bases A1, C11 and T12 as observed in the experimental structures. A1 is colored purple, and C11 and T12 are in yellow and green, respectively. (**a**) In the NMR structure, (**b**) chain A in the 1.62-Å ^Br^U c-KIT crystal structure, (**c**) chain B in the 1.62-Å ^Br^U c-KIT crystal structure, (**d**) chain A in the 1.82-Å trigonal crystal structure, (**e**) chain B in the 1.82-Å trigonal crystal structure, (**f**) chain A in the 2.73-Å hexagonal crystal structure, (**g**) chain B in the 2.73-Å hexagonal crystal.

The bases at the dimer stacking interfaces (A1, C11 and T12) show the most striking difference between the four individual c-KIT G-quadruplexes, the ones reported in the earlier ^Br^U crystal structure, and the NMR structure (Figures 3a and b and 4). In the NMR c-KIT structure, the two bottom bases A1 and T12 form a Watson–Crick base pair and stack onto the bottom quartet surface (Figure 4a). In the earlier ^Br^U (orthorhombic) G4 crystal structure, there is hydrogen bonding in chain A between N6 of A1 and O2 of ^Br^U12, and a water molecule bridges between N1 of A1 and N3 of ^Br^U12 (Figure 4b). A1 of the ^Br^U G4 in chain B is oriented away of the structure, and bases C11 and ^Br^U12 are stacking on the quartet surface, beneath the contact between a potassium ion and a water molecule (Figure 4c).

In the two new c-KIT crystal structures, two quadruplexes stack each other through the bottom G-quartet, and the interface bases A1, C11 and T12 are oriented away from the common surface (Figure 4d and e). The two A1 bases in each chain asymmetric unit form a stacking pair in both two crystal structures, while C11 and T12 have different roles. In the trigonal crystal form, C11 and A5 from different asymmetric units form a long stacking unit. Two C11 bases in the 2.7-Å hexagonal crystal structure form a hydrogen bond with a G7 phosphate oxygen atom in the other asymmetric unit, and the T12 base stacks with the G18 base from another asymmetric unit.

These differences between the total of six independent c-KIT quadruplexes in the three crystal structures are also seen in the diverse conformations of several others of the external, non-hydrogen-bonded bases (Figure 3a and b). Backbone torsion angles for the extra-helical nucleotides adopt a wide range of values (see Table 2 and Supplementary Table S1), with very few torsion angles being conserved across the six independent quadruplex chains (values for the nucleotides comprising the stacked G-quartet core are in general highly conserved–see the Supplementary data for a full listing of torsion angles). Loop angles δ and to a lesser extent ζ have conserved values, the most notable exception being in the quadruplex A chain of the 2.73-Å crystal structure. In the ^Br^U form, bases A1, A5 and C9 are directed out from the quadruplex core, base C11 from chain B is tucked into the core, under the G10 deoxyribose, and the ^Br^U at position 12 is also tucked in, partially stacking onto G13. In the high-resolution 1.82-Å structure bases A1, A5 and C9 are also oriented outward, as is C11. Base T12 however adopts a distinct, partially swung-out conformation, with the edge of the thymine coming closer to the backbone at G22. By contrast, these bases in the ensemble of NMR structures adopt a significantly narrower range of conformations (Figure 3c).

**Table 2.** The backbone torsion angles for the quadruplex loop residues of the NMR structure (model 1 only) and the three crystal structures

		NMR	^Br^U c-KIT 1.62 Å		c-KIT 1.82 Å		c-KIT 2.73 Å	
			Quad-A	Quad-B	Quad-A	Quad-B	Quad-A	Quad-B
A1	γ	−63	−63	−74	−125	−118	−142	−57
	δ	118	157	131	91	149	101	91
	ϵ	180	−76	−155	74	−101	104	55
	ζ	−81	−158	63	−155	−64	−145	142
	χ	39	−100	−80	−108	−136	−107	−72
A5	α	52	59	−89	71	81	119	80
	β	128	154	−169	152	168	179	150
	γ	55	45	168	48	26	−29	31
	δ	149	149	110	93	128	125	109
	ϵ	179	−119	−96	−150	−151	−162	−145
	ζ	156	83	160	−169	173	−163	176
	χ	−51	−153	−159	−133	−81	−87	−97
C9	α	75	57	66	72	69	64	−90
	β	176	155	140	169	156	135	−101
	γ	59	49	57	50	52	56	158
	δ	131	107	106	145	98	104	76
	ϵ	−108	−153	−143	−125	−155	−129	−115
	ζ	−149	−172	169	112	−172	158	152
	χ	−128	−155	−100	−160	−113	−161	−140
C11	α	10	45	151	50	53	−101	−54
	β	128	98	−143	−160	−176	−120	−128
	γ	−48	175	50	67	70	161	127
	δ	135	152	151	150	142	106	82
	ϵ	−144	−99	−140	−163	−151	−118	−166
	ζ	−127	−79	−170	149	161	143	−134
	χ	−175	29	−170	−128	−146	−114	−176
T12	α	−106	−74	−80	−138	−166	74	−114
	β	−103	−163	−157	−146	−125	102	−131
	γ	63	52	56	68	68	−7.1	77
	δ	145	111	151	141	144	85.2	78
	ϵ	173	−150	−173	−163	−134	36.6	62
	ζ	−104	−123	−76	133	82	158.4	−105
	χ	−121	−156	−133	−126.	−112	25.2	13

The most pronounced difference between the crystal structures and the NMR models involves the large cleft between the stem-loop (G18, A19 and G20) and the adjacent G-quartet. The net effect of these differences is that the large cleft is consistently narrower in all of the crystal structures.

### MD simulations

Multiple-trajectory MD simulations of the intramolecular 22-mer c-KIT G4 in five distinct systems were carried out for 250 ns, each repeated in triplicate, for a total of 3750-ns simulation time. The systems were:

#1: chain A (with 4 K^+^ and 2 Mg^2+^ ions) of the ^Br^U 1.62 Å c-KIT G4
#2: chain B (with 4 K^+^ ions) of the ^Br^U 1.62 Å c-KIT G4
#3: chain A (with 3 K^+^ ions) of the 1.82 Å c-KIT G4
#4: chain B (with 2 K^+^ ions) of the 1.82 Å c-KIT G4
#5: model 1 (with 2 K^+^ ions) from the c-KIT G4 NMR structure

**Figure 5.**
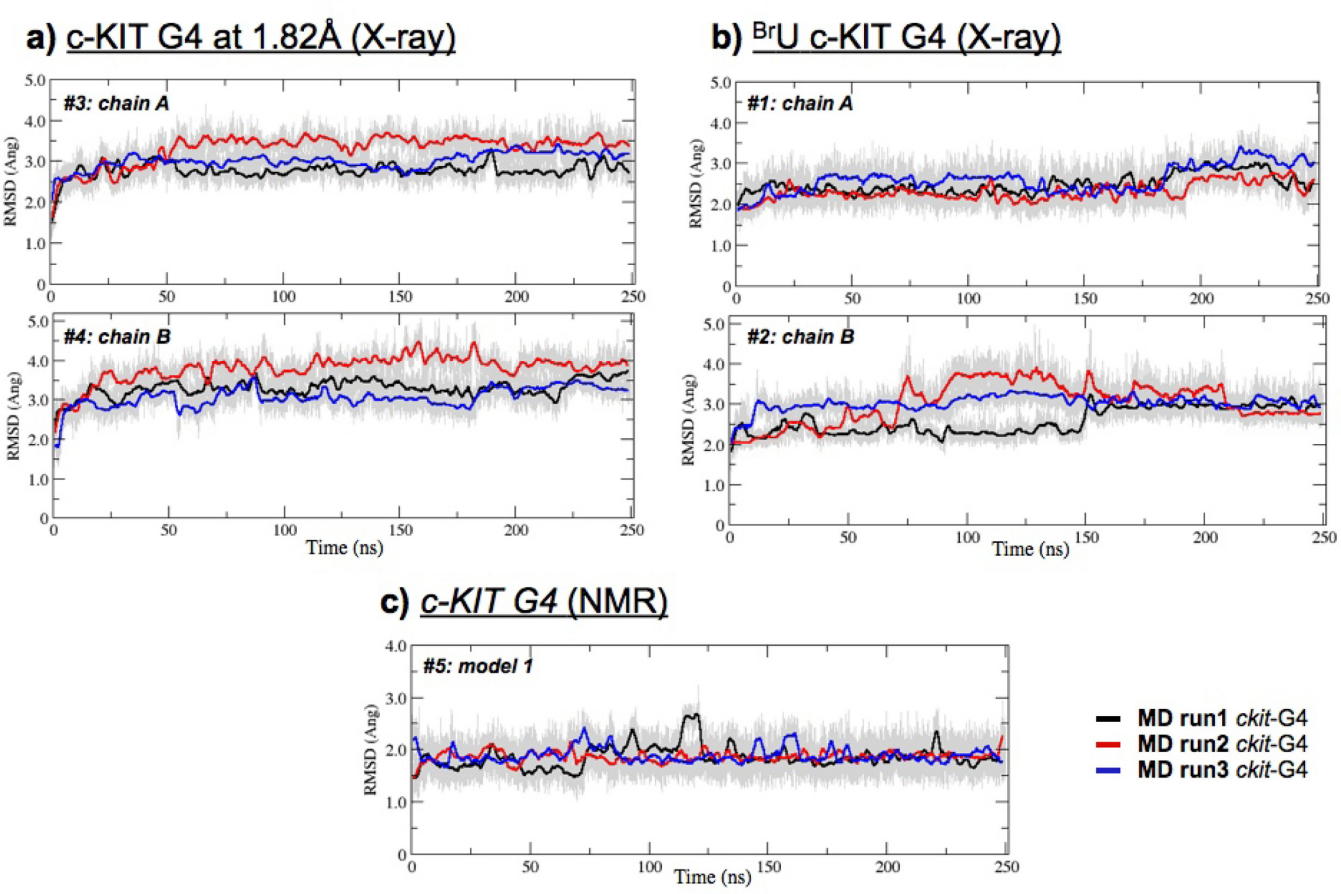
RMSD plots showing the structural convergence of the simulated systems during multiple MD runs of the c-KIT 22-mer G-quadruplexes. Each of the 250-ns MD runs was performed three times, for all five simulated systems: chains A and B of the native c-KIT (X-ray) (**a**) chains A and B of the ^Br^U c-KIT X-ray structure, pdb 3QXR (**b**) model 1 of the c-KIT NMR structure, pdb 2O3M (**c**) Running averages over 500 frames of the RMSD plots (gray) for MD run 1 (black), MD run 2 (red) and MD run 3 (blue).

The multiple-trajectory approach enabled improved sampling of the conformational space to be obtained for a given set of conditions/local minima, resulting in more statistically robust conclusions than analyses based on a single trajectory approach. The structural convergence of the five systems over the course of the multiple 250 ns simulations is graphically represented as RMSD plots in Figure 5 and summarized in Supplementary Table S2. Table 3 summarizes the ions and water molecules in the simulations. In the absence of the structural central channel ions that were manually placed into the NMR model prior to starting the simulations (#5), the system collapsed. (Data not shown here, but during preliminary 250-ns simulations the NMR structure became unwound and unstable in the absence of K^+^ ions).

**Table 3.** Listing of the ions and water molecules in the simulations

System	Timescale	Residues	Ions		TIP3P waters	
			Structural	Counter-ions	Structural	Total
#1 ^Br^U c-KIT G4 chain A	3 × 250 ns	22 bases	4 K^+^, 2 Mg^2+^	13 K+	123	4900
#2 ^Br^U c-KIT G4 chain B	3 × 250 ns	22 bases	4 K^+^	17 K+	88	6176
#3 1.82-Å c-KIT G4 chain A	3 × 250 ns	22 bases	3 K^+^	18 K+	164	6092
#4 1.82-Å c-KIT G4 chain B	3 × 250 ns	22 bases	2 K^+^	19 K+	164	6096
#5 model 1 c-KIT G4 NMR	3 × 250 ns	22 bases	2 K^+^	19 K+	/	4775

**Figure 6.**
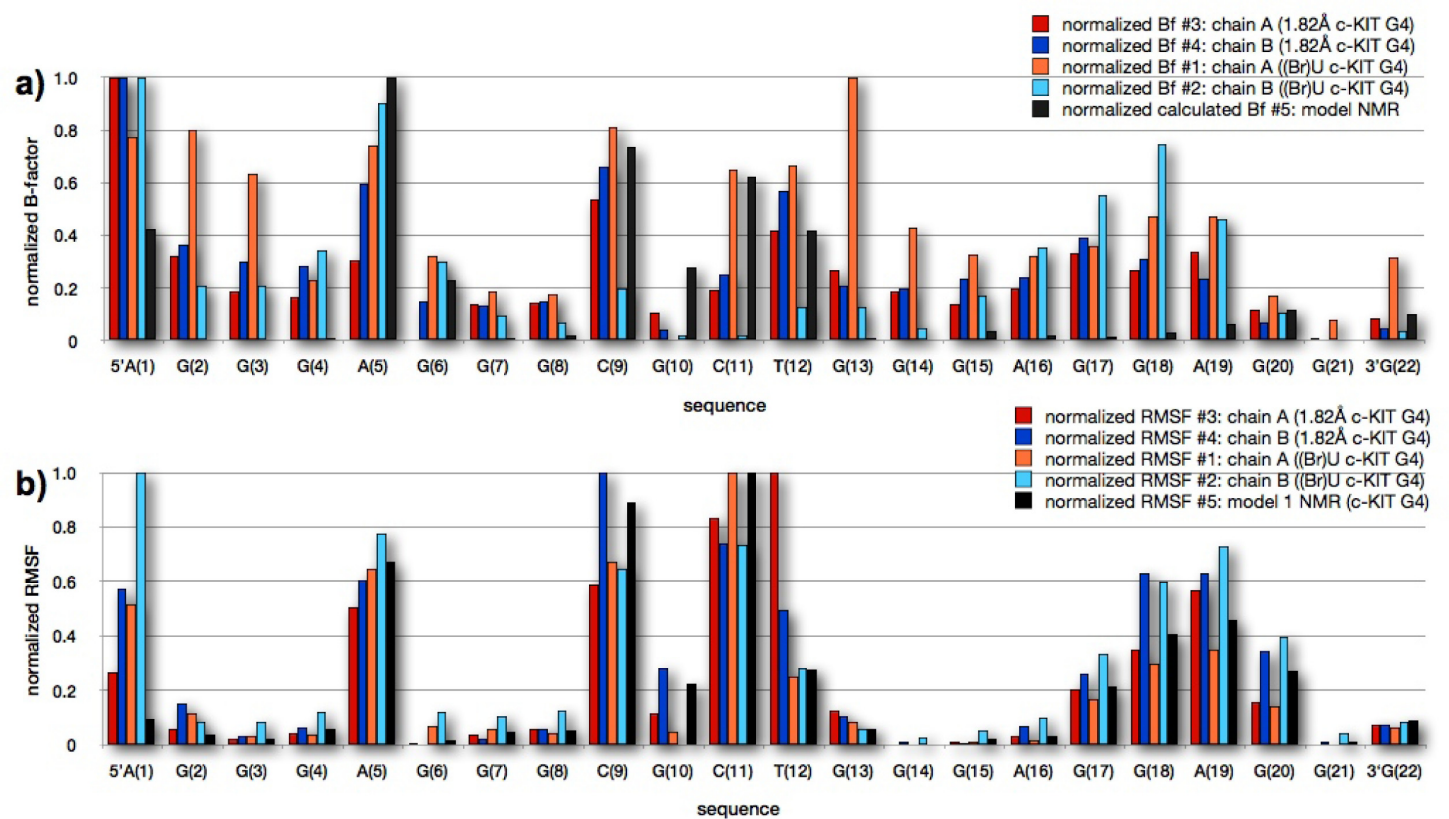
(**a**) Plot showing the normalized B-factor values from the experimental crystal structures, on a per-residue basis. (**b**) RMS fluctuations from the MD simulations, calculated on a per-residue basis for the five c-KIT G4 systems studied. #1 G4 and #2 G4 from the native c-KIT G4 crystal structure are shown in red and blue, respectively, #3 G4 and #4 G4 from the ^Br^U c-KIT G4 are shown in orange and cyan, respectively, and #5, the c-KIT G4 NMR structure is shown in black.

The flexibility of the bases within these systems was explored prior to starting the MD simulations, by plotting the averaged normalized B factor values per residue that were obtained from the X-ray structures (Figure 6a). Although experimental B factors are purely crystallographic quantities, theoretical B-factors (per residue) were also calculated, and normalized, for the c-KIT G4 NMR structure, employing all the conformations within the NMR bundle. These theoretical B factors were calculated by means of the GROMACS g_rmsf tool. This shows a consistent pattern of increased B values for those nucleotides with extra-helical bases, especially at and around A1, A5, C9, C11 and T12. Nucleotide A19 is also flexible, even though the adenine base is stacked onto G18. The RMSD values averaged per residue over all of the models within the NMR structure were calculated and normalized by means of the GROMACS *g_rmsf* tool (Figure 6a). Comparison of the crystallographic and NMR-derived fluctuations shows that the flexibility of residues A5, C9, C11, T12 and A19 is apparent both in the experimental and simulated structures. The RMS fluctuations of individual bases were calculated on completion of the MD simulations from all three trajectories available for each of the five systems, and were normalized (Figure 6b). There is consistently good agreement between the experimental and simulation data, with the same regions and nucleotides in all of the c-KIT quadruplexes showing equivalent flexibility and stability.

**Figure 7.**
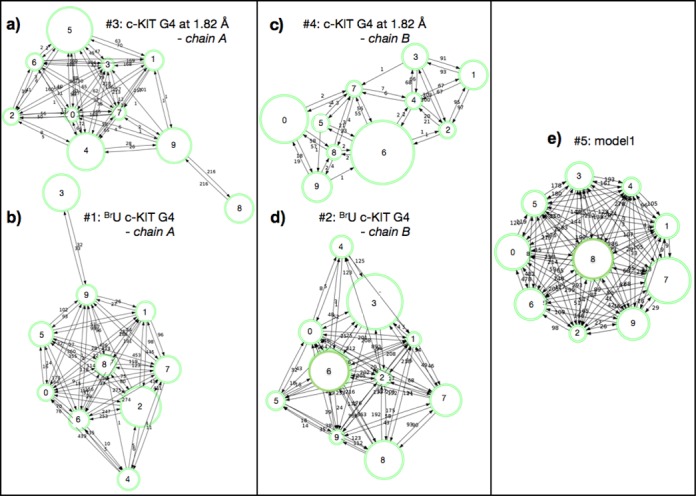
Clusters of c-KIT G4 conformations sampled through the multiple MD runs. The triplicate MD trajectories (i.e. 750 ns) of the two c-KIT G4 chain A were clustered by the robust clustering algorithm to yield (**a**) 10 clusters of #3 G4, (**b**) 10 clusters of #1 G4, (**c**) 10 clusters of #4 G4, (**d**) 10 clusters of #2 G4 and (**e**) 10 clusters of #5 G4, in order to identify the predominant conformations sampled throughout the simulations. In the graphical representation of the clusters (a–e) transitions between individual clusters were counted and indicated by arrows. The cluster sizes (i.e. the number of structures within a cluster) are represented by circles.

**Figure 8.**
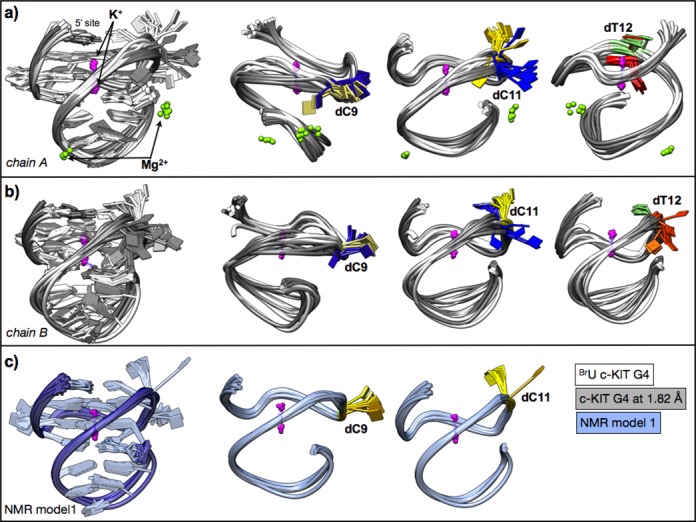
Conformations of the c-KIT G4 conformations sampled through the multiple MD runs. (**a**) Upon superimposition of all 20 c-KIT G4 chain A cluster-representative medoid structures (10 from #3 G4 and 10 from #1 G4), the highest level of conformational flexibility was found for bases C9, C11 (#3 G4 in blue, #1 G4 in yellow) and T12 (#3 G4 in orange, #1 G4 in green). (**b**) All c-KIT G4 chain B cluster-representative medoid structures (i.e. 20 conformations) were superimposed and compared; bases C9, C11 (#4 G4 in blue, (#2 G4 in yellow) and T12 (#4 G4 in orange, #2 G4 in green) displayed the highest level of conformational variability (base flipping). (**c**) The superposition of the NMR-based c-KIT conformations (#5 G4, 10 conformations) shows base flipping at bases C9 and C11 (yellow).

The three 250-ns MD runs per system were clustered together, resulting in 10 clusters per system. Each of the identified clusters is represented by a medoid structure (i.e. a representative structure/object of the cluster whose average dissimilarity to all other structures/trajectory frames in the cluster is minimal). Each of the medoids corresponds to a physical frame from the trajectory. The probability of formation (i.e. the cluster population) of each cluster was also calculated and is reflected in the graphical representation of the clusters (Figures 7 and 8).

Although clustering was performed solely for the nucleic acid atoms, the complete systems—including the structural ions—were retrieved for visualization. The structural K^+^ ions within the central core in between the quartets always remained at their respective positions (Figures 7 and 8), as well as the Mg^2+^ ions, which remained within the loops of the #1 G4 structure (Figure 7). However the K^+^ ions initially present in the loops of systems #1, #2 and #3 did not remain at their sites throughout the course of the MD runs.

Clusters of chains #1 and #3 (Figure 7) from both X-ray structures displayed a common pattern. Nine of the 10 clusters are highly interconnected, i.e. undergoing multiple transitions between structural conformations, but one cluster is isolated (cluster no. 8 for #3, and cluster no. 3 for #1; the population in both cases ranges between 12 and 15%, respectively), and is only connected to one of the other clusters. Superimposition of all the 20 representative structures of #1 and #3 shows that their conformations are overall in good agreement, with the exception of bases C9, C11 and T12 where significant base flipping occurs (Figure 7).

Superimposition of the cluster representatives (medoid structures) of #2 and #4 G4s reveals more conformational flexibility/variability compared to #1, in particular in the loop region formed by bases A16-G20. Clear base-flipping occurs at the corresponding bases C9, C11 and T12 (Figure 7).

In terms of medoid structures obtained from the NMR model MD simulations (Figure 8), very good agreement was observed for the conformations, in accord with high stability of the simulation data (trajectories). The clusters are also very well interconnected with high frequencies of transitions between them (Figure 8a). Bases C9 and C11 were the only two where some conformational variability of the bases was found (Figure 8b). However that was to a much smaller extent compared to the X-ray structures.

**Figure 9.**
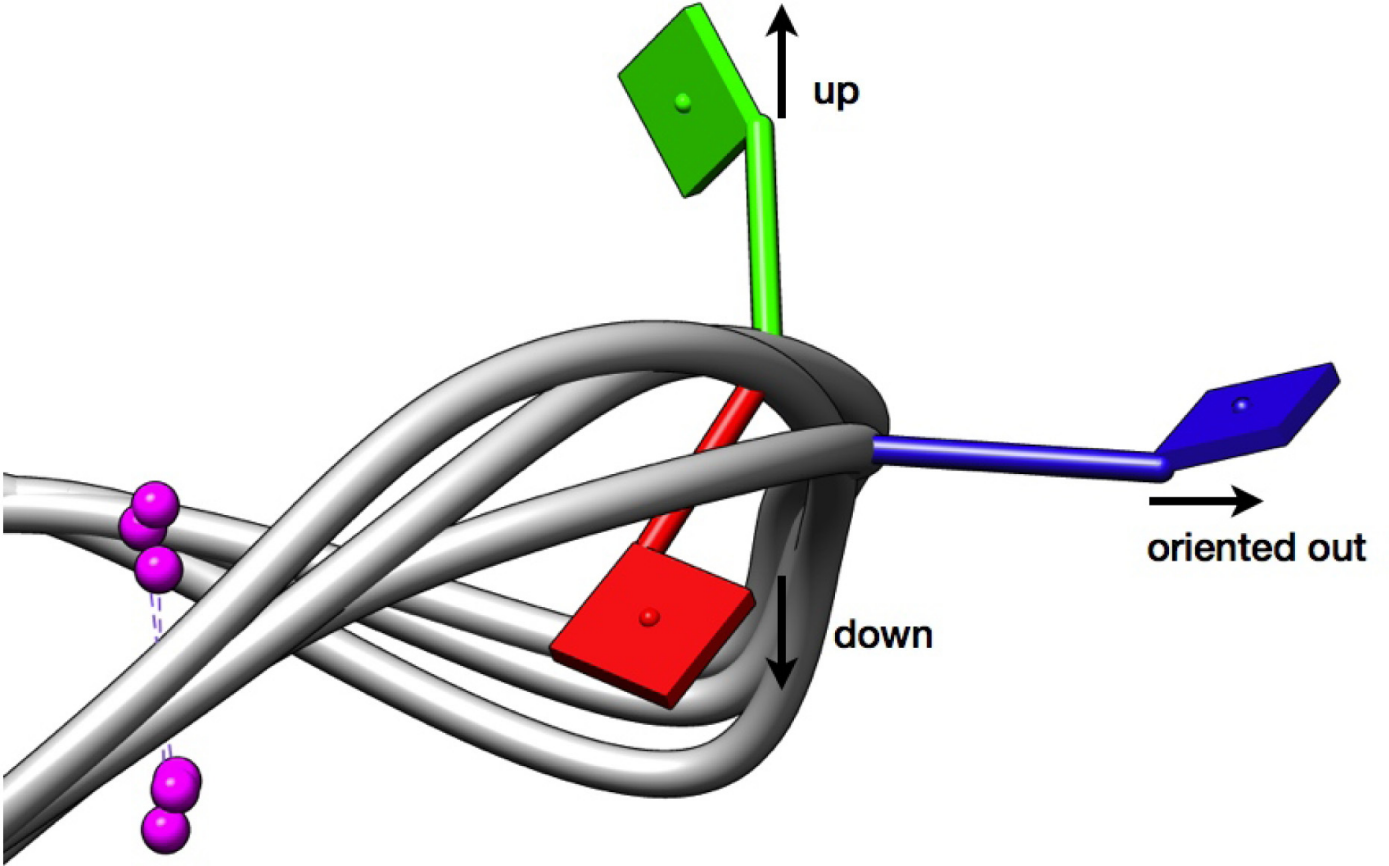
Different positions/orientations of bases identified in cluster representatives sampled throughout the MD runs. Bases C9, C11 and T12 were found to be oriented up (green), down (red) and away from the backbone (blue). The bases were also found to be stacking on the bases of other G-quartets (not shown here).

The flipping (and change of orientation) of bases C9, C11 and T12, determined from the clustering data of all five systems, was further examined and quantified, and is summarized in Table 4. Visualizing/keeping the G-quartets in plane, orthogonal to the K^+^ ions in between the G-quartets, has enabled four orientations of these bases to be identified with respect to the DNA backbone: (i) up, (ii) down, (iii) oriented out, (iv) stacking, as shown in Figure 9. These positions correspond to those described in Table 4.

**Table 4.** Overview of the conformational changes of bases C9, C11 and T12 sampled throughout the three repetitive MD runs of each of the five studied systems

Base	Chain A		Chain B		#5 model 1 c-KIT G4 NMR
Orientation	#1 ^Br^U c-KIT G4	#3 1.82-Å c-KIT G4	#2 ^Br^U c-KIT G4	#4 1.82-Å c-KIT G4	
C9 →	85%	93%	100%	74%	100%
C9 ↑	-	-	-	15% (cl. 2, 3)	
C9 ↓	15% (cl. 0, 4)	7% (cl. 2)	-	11% (cl. 9)	
C11 →	8% (cl. 9)	73%	-	62% (cl. 0, 6–9)	16% (cl. 7)
C11 ↑	77%	15% (cl. 9)	100%	32% stack on G10	84%
C11 ↓	15% (cl. 3)	12% (cl. 8)	-	6% (cl. 5)	-
T12 →	-	16% (cl.4)	-	24% (cl. 6)	-
T12 ↑	-	-	-		-
T12 ↓	-	43%	-	76%	-
T12 stack (G13)	100%	27% (cl. 8, 9)	100%	-	100%

Base C9 remained predominantly in the same orientation in all five simulated systems, as in all three experimental structures: it is oriented away from the DNA backbone. This base did sometimes adopt other orientations, although not in structure #2 or the NMR model. Base C11 has the highest degree of conformational variability, in both #3 and #4 G4s, as well as #1. T12, on the other hand, was found to be stacking on base G13 in the NMR model as well as in both #1 and #2 structures. In #3 and #4, T12 was also found to be oriented away from the DNA backbone.

## DISCUSSION

### The short c-KIT loops are very flexible

The consistent experimental observations of only very limited conformational flexibility for the core of the c-KIT quadruplex in all the crystal and NMR structures are in overall excellent accord with the MD data, as shown in particular in Figure 6, comparing experimental B factors with calculated RSMD values. Similarly the conclusions from the simulations that the loop nucleotides on the exterior of the structures are conformationally flexible and show extensive base flipping are in good accord with the experimental observations from the three separate crystal structures and the NMR structure.

The trinucleotide loops in human telomeric quadruplexes have been extensively explored by MD simulation methods (57–63) and these have also been found to be conformationally mobile (60,63). Although a range of conformations have been observed in these simulations, dependent on loop type, base–base stacking in the loops does stabilize some of them for at least part of the simulation time. Loop flexibility has also been reported in a previous c-KIT quadruplex simulation (64), and the present study extends this finding by showing that single or dinucleotide loops are less likely to form discrete transiently stabilized base-stacked loops than are trinucleotide ones, as in human telomeric quadruplexes. By contrast the pentanucleotide AGGAG loop in the c-KIT quadruplexes is rather stable, with again experiment and simulation in broad agreement.

### The potential ligand binding sites of the c-KIT quadruplex

The flexibility of the external unpaired bases, in particular those forming the two single-nucleotide loops A5 and C9 and the dinucleotide loop C11, T12, is also of potential significance for small-molecule binding to the termini of the c-KIT quadruplex. For example, one site may involve the stacking of T12 onto the bottom G-quartet, as observed in the ^Br^U c-KIT crystal structure and the NMR structure. This feature also has 27% occurrence in the simulated chain A of the 1.82 Å c-KIT G4 trigonal form (Table 4), even though the stacking is not found in the initial starting point for this particular structure. Some ligands may stack on top of the *in situ* T12-A1 stack rather than onto this terminal G-quartet, which would involve displacing the T12-A1 base pair.

The large cleft in the c-KIT quadruplex is conserved in all the structures, experimental and theoretical, although its dimensions do have some variability (see, in particular, Figure 8). The cleft diameter can be defined by P…P inter-strand separation distances across the cleft. Distance P(G8)…P(G20) is 14.8 Å in the NMR structure, 8.1 Å in the ^Br^U crystal structure and 8.7 Å in the 1.82 Å crystal structure reported here. The cleft is of sufficient size and nature to be a suitable small-molecule binding site. Even though *in silico* docking methods need to take the flexibility of cleft dimensions into account, the nature of the site is well-suited to experimental fragment-based approaches (53,65) to discover novel c-KITquadruplex-selective small molecules capable of down-regulating c-KIT expression.

### The c-KIT quadruplex core topology is conserved

Quadruplex DNA and RNA nucleic acids can in principle adopt a wide range of topologies, and X-ray and NMR methods have to date almost certainly only accessed a small cohort of them. The potential for a large, but as yet undefined number of folds is due to several factors, including (i) the inequality of G-tract lengths in many quadruplexes coupled with (ii) the diversity of loop lengths and sequence. It is apparent, for example, that when a guanosine nucleotide occurs in a loop region, it can become directly involved in quartet formation and thus induces more complex arrangements than is the case with simple quadruplex-forming sequences containing non-guanosine loops. The c-KIT quadruplexes is an exemplar of this complexity of structure.

The present study addresses the question of whether the c-KIT quadruplex shows conformational diversity in differing environments. The earlier observations were that its fold as found by NMR in solution was also observed in the crystalline state. These findings have been reinforced by the determination of the two further c-KIT crystal structures reported here, each of which occurs in a distinct space group. These thus in total provide visualizations of the c-KIT quadruplex in three distinct crystal lattice environments. The differing lattices have not imposed restrictions on the conformations of those external bases of the c-KIT quadruplex, which are unencumbered by hydrogen bonding or internal stacking. Instead these bases are found in a diversity of conformations, comparable to those in the NMR ensemble (compare Figure 3b and c). The CD spectrum of the c-KIT DNA quadruplex in K^+^ solution shows the features of a typical parallel folded quadruplex (data not shown), with a prominent positive signal at around 260 nm and a negative signal at around 240 nm, fully consistent with the fold observed in the crystallographic and NMR c-KIT quadruplex structures.

It can thus be concluded that the core fold of the c-KIT quadruplex is highly conserved, stable and is not environmentally sensitive, at least in K^+^ conditions. This is in striking contrast to the high topological variability shown by human telomeric quadruplex sequences (see, for example (66–76)). This suggests that topological variability is not inherent to quadruplexes as a general class of nucleic acid structures. Rather, only those quadruplexes that have loops containing at least two nucleotides can readily show such variability since single-nucleotides are generally constrained to form propeller loops and are unable to refold to form other types of loop, for stereochemical reasons (a single nucleotide is of insufficient dimensions to form a diagonal or lateral loop). The findings here are in accord with single-molecule studies of the c-KIT quadruplex (77) embedded in a duplex, which show that its dynamics are much reduced compared to the human telomeric quadruplex, and reinforce the view that the stability of the c-KIT topology makes it a suitable druggable quadruplex target for small molecules. The majority of promoter and other quadruplexes for which there is experimental folding data, from CD studies and in some instances from NMR studies, have at least one single-nucleotide loop and parallel folds have mostly been assigned to these structures (see, for example (24,^30^,^78^–^81^)). We speculate from this that intramolecular quadruplexes can be grouped into two broad classes (i) those with at least one single-nucleotide loop, which often show singular topologies even though loops are highly flexible, and (ii) with all loops comprising at least two nucleotides, which is likely to lead to folding dynamism, and with loops having more stable and less dynamic base-stacked secondary structures.

## SUPPLEMENTARY DATA

Supplementary Data are available at NAR Online.

SUPPLEMENTARY DATA
