# Supplementary material for: Flexibility and structural conservation in a c-KIT G-quadruplex
Source: Nucleic Acids Res. 2014 Dec 1;43(1):629–44. doi: 10.1093/nar/gku1282 (PMC4288176; doi:10.1093/nar/gku1282)
Supplement: SUPPLEMENTARY DATA [file supp_43_1_629__index.html]

Flexibility and structural conservation in a c-KIT G-quadruplex — Flexibility and structural conservation in a c-KIT G-quadruplex — SUPPLEMENTARY DATA 

# Flexibility and structural conservation in a c-KIT G-quadruplex

## SUPPLEMENTARY DATA

**Files in this Data Supplement:**

- SUPPLEMENTARY DATA
