# Supplementary material for: Flexibility and structural conservation in a c-KIT G-quadruplex
Source: Nucleic Acids Res. 2014 Dec 1;43(1):629–44. doi: 10.1093/nar/gku1282 (PMC4288176; doi:10.1093/nar/gku1282)
Supplement: SUPPLEMENTARY DATA [file supp_gku1282_nar-02998-f-2014-File002.docx]

Supplementary Data

**Flexibility and structural conservation in a c-KIT G-quadruplex**

**Dengguo Wei^1^ Jarmila Husby^2^ and Stephen Neidle***

UCL School of Pharmacy, University College London, London WC1N 1AX, UK

^1^ Now at: Department of Chemistry, Umeå University, Umeå SE-901 87, Sweden

^2^ now at: Istituto Italiano di Tecnologia, Genova, Italy

For clustering of the MD data, a robust algorithm (56) combining a k-medoids algorithm with an effective cluster initialization (55) was used. There are a number of advantages offered by that clustering technique, including (1) direct minimization of a functional which has a clear geometric meaning, (2) direct delivery of the centroids as frames belonging to the trajectory and (3) good partitioning of the samples due to an effective initialization procedure (yielding reproducible clustering results independent of the frame order). The clustering metric is based on the RMSD of the heavy atoms belonging to the binding site (if specified), and in order to avoid prioritizing any particular frame configuration, the RMSD is calculated for all the possible frame pairs. The final number of clusters was manually selected to 10, in order to avoid over-fitting, and to obtain easily intelligible data.

**Table S1**. The backbone torsion angles of the quadruplexes in the NMR structure (model 1 only) and the three crystal structures.

|  |  | NMR |  | ^Br^U c-KIT 1.62 Å | |  | c-KIT 1.83 Å | |  | c-KIT 2.873Å | |
| --- | --- | --- | --- | --- | --- | --- | --- | --- | --- | --- | --- |
|  |  |  |  | Quad-A | Quad-B |  | Quad-A | Quad-B |  | Quad-A | Quad-B |
| A1 | χ | 39.1 |  | -100 | -80.2 |  | -108.4 | -135.7 |  | -74.8 | -114 |
|  | δ | 118.3 |  | 157.1 | 130.9 |  | 91.2 | 149.2 |  | 27.3 | 20.8 |
|  | ε | 179.7 |  | -76.4 | -154.8 |  | 73.8 | -100.7 |  | 79.5 | 115.1 |
|  | γ | -63 |  | -62.8 | -73.8 |  | -125.2 | -117.5 |  | 62.6 | 152.3 |
|  | ζ | -80.6 |  | -158.2 | 63.5 |  | -154.5 | -64.4 |  | 126.8 | -150.4 |
| G2 | α | -69.6 |  | 64.8 | 63 |  | -50 | -67.1 |  | -75.6 | 46.8 |
|  | β | -169.7 |  | -153.7 | 101.5 |  | 135.4 | -121.1 |  | -152.9 | -169.3 |
|  | γ | 50.6 |  | 178.9 | -178.1 |  | 6.8 | 54.5 |  | 34.8 | 78.1 |
|  | δ | 137.5 |  | 143.5 | 82.3 |  | 121.1 | 141 |  | 153.1 | 145.7 |
|  | ε | 163 |  | -128.2 | -158.9 |  | -174 | -171.5 |  | -175.7 | -135.9 |
|  | ζ | -110.1 |  | -163.3 | -69.1 |  | -91.1 | -74.5 |  | -89.7 | -126 |
|  | χ | -96.6 |  | -112 | -169 |  | -134.5 | -133.4 |  | -116.8 | -131.4 |
| G3 | α | -58.2 |  | 70.9 | -65.9 |  | 155.5 | 169.2 |  | 160 | 84.7 |
|  | β | 179.7 |  | -152.2 | -168.7 |  | 155.4 | 135.5 |  | 158.2 | -148 |
|  | γ | 56.1 |  | -142.1 | 58 |  | 176.4 | 162.1 |  | 169.3 | -142.7 |
|  | δ | 140.2 |  | 143.4 | 148.7 |  | 145.6 | 143.8 |  | 143.5 | 134.3 |
|  | ε | 163.7 |  | -171.8 | -175.9 |  | -168.5 | -168.2 |  | -173.3 | -162.3 |
|  | ζ | -90.1 |  | -106.2 | -102.5 |  | -98 | -93.2 |  | -96 | -96.7 |
|  | χ | -116.2 |  | -139.5 | -129.1 |  | -136.8 | -140.4 |  | -131.8 | -146.6 |
| G4 | α | -70.8 |  | -71.1 | -62.1 |  | -71.1 | -77.3 |  | -73.6 | -63.7 |
|  | β | -160.7 |  | -167.7 | -159.6 |  | -163.6 | -161.4 |  | -142.7 | -156.2 |
|  | γ | 50.2 |  | 60.6 | 39.3 |  | 46.9 | 49 |  | 35.3 | 30.8 |
|  | δ | 137 |  | 150.5 | 149.6 |  | 144.6 | 146.3 |  | 156.5 | 154.2 |
|  | ε | -59.8 |  | -72.9 | -93 |  | -78.7 | -78 |  | -62 | -73.2 |
|  | ζ | 77.2 |  | 84.5 | 109.4 |  | 86.4 | 86 |  | 68.6 | 89.8 |
|  | χ | -116.2 |  | -119.2 | -110.9 |  | -106.1 | -102.3 |  | -89.8 | -94.9 |
| A5 | α | 52.2 |  | 59.2 | -89 |  | 71.3 | 80.7 |  | 83.3 | 120.5 |
|  | β | 128 |  | 153.5 | -169.2 |  | 152.4 | 167.6 |  | 139.1 | 176.1 |
|  | γ | 54.6 |  | 45.1 | 167.8 |  | 48.1 | 25.8 |  | 35.6 | -30 |
|  | δ | 149.3 |  | 149.2 | 109.6 |  | 92.6 | 128.2 |  | 99.8 | 121.8 |
|  | ε | 179.1 |  | -119.3 | -95.6 |  | -149.9 | -151.1 |  | -143.7 | -168.6 |
|  | ζ | 156.3 |  | 83.1 | 159.5 |  | -169.1 | 172.7 |  | -179.8 | -159 |
|  | χ | -51.2 |  | -153 | -159.1 |  | -133.5 | -81 |  | -97.8 | -86.7 |
| G6 | α | -80 |  | 105.7 | 35.6 |  | 59.3 | 52.6 |  | 73.3 | 79.8 |
|  | β | 125.5 |  | -156 | -154.7 |  | -128 | -124 |  | -120.9 | -126.5 |
|  | γ | -64.3 |  | 13.4 | 44.1 |  | 53.3 | 55.2 |  | 36.8 | 44.3 |
|  | δ | 127.6 |  | 88.9 | 93.8 |  | 141.3 | 142.1 |  | 154.3 | 152.1 |
|  | ε | -178.2 |  | -165.5 | -177.8 |  | -163.5 | -165.8 |  | -164.6 | -161.6 |
|  | ζ | -87 |  | -85.7 | -84.3 |  | -92.7 | -98.4 |  | -100.7 | -100.2 |
|  | χ | -125.5 |  | -165.2 | -154.2 |  | -129.6 | -125.9 |  | -116.5 | -124.9 |
| G7 | α | -77.9 |  | -57.3 | -56.7 |  | -92.8 | -83.2 |  | -93.4 | -89.7 |
|  | β | 176.9 |  | -179.5 | -175.6 |  | -177 | -174.4 |  | -172.3 | -179.2 |
|  | γ | 60.7 |  | 62.5 | 49 |  | 62.8 | 56.9 |  | 59.6 | 61.6 |
|  | δ | 123.7 |  | 143.8 | 142 |  | 124.9 | 127.9 |  | 131.7 | 121.5 |
|  | ε | 174 |  | 176 | -177.3 |  | 170.1 | 167.2 |  | 170.9 | 167 |
|  | ζ | -105.6 |  | -106.3 | -101.2 |  | -99.4 | -96.1 |  | -138.8 | -99.5 |
|  | χ | -124.4 |  | -111.6 | -126.7 |  | -114.4 | -113 |  | -101.5 | -109.7 |
| G8 | α | -78 |  | -69.2 | -65.1 |  | -52.9 | -68.3 |  | 13.2 | -44.5 |
|  | β | -169.8 |  | -163.9 | -167.2 |  | -160.8 | -158.5 |  | 152.3 | -155.1 |
|  | γ | 60.5 |  | 53.1 | 50.8 |  | 38.2 | 49.1 |  | 7.6 | 28.3 |
|  | δ | 125.8 |  | 139.1 | 143.3 |  | 142.3 | 140.5 |  | 153.9 | 151.7 |
|  | ε | -69.3 |  | -70.1 | -73.9 |  | -83.7 | -82.1 |  | -69.6 | -77.4 |
|  | ζ | 81.6 |  | 97.2 | 89.2 |  | 81.7 | 88.6 |  | 113.8 | 80.6 |
|  | χ | -130.9 |  | -111.8 | -120.9 |  | -109.3 | -111.2 |  | -98.2 | -100.8 |
| C9 | α | 74.6 |  | 57.3 | 65.9 |  | 71.8 | 69.4 |  | -91.8 | 60.4 |
|  | β | 176.2 |  | 154.9 | 140.2 |  | 168.6 | 156.4 |  | -101.9 | 135.9 |
|  | γ | 58.8 |  | 49.3 | 57.3 |  | 50.4 | 51.8 |  | 159.9 | 57.9 |
|  | δ | 131 |  | 106.5 | 106.2 |  | 144.7 | 97.7 |  | 79.3 | 103.1 |
|  | ε | -108.2 |  | -152.6 | -142.6 |  | -125 | -155.3 |  | -111.1 | -124.5 |
|  | ζ | -148.7 |  | -172.4 | 168.9 |  | 111.9 | -171.7 |  | 151 | 158.9 |
|  | χ | -127.6 |  | -155.1 | -100.3 |  | -157.9 | -112.7 |  | -141.5 | -164.4 |
| G10 | α | -53.2 |  | 67.9 | 61.6 |  | 66.8 | 50.2 |  | 65.7 | 61.1 |
|  | β | -115.7 |  | -137.2 | -138.4 |  | 177.6 | -141.9 |  | -175.9 | -147.2 |
|  | γ | 60.5 |  | 59.9 | 58.3 |  | 57.6 | 72.5 |  | 57.8 | 61.3 |
|  | δ | 149.4 |  | 147.5 | 161.1 |  | 145.5 | 147.7 |  | 140.3 | 146.7 |
|  | ε | 149.7 |  | -80.7 | -54.6 |  | -108 | -114.7 |  | -116.3 | -147.5 |
|  | ζ | -164.4 |  | 100.5 | 90.8 |  | -136.9 | -133.5 |  | -92.5 | -102.4 |
|  | χ | -152.2 |  | -124.9 | -124 |  | -134.3 | -136.8 |  | -134.6 | -131.6 |
| C11 | α | 9.5 |  | 45.3 | 150.6 |  | 49.7 | 53.1 |  | -57.5 | -97.4 |
|  | β | 127.8 |  | 98 | -143.3 |  | -160.2 | -176 |  | -118.1 | -117.2 |
|  | γ | -48.1 |  | 174.9 | 49.6 |  | 66.5 | 70.3 |  | 126.7 | 159.6 |
|  | δ | 135 |  | 152.3 | 150.5 |  | 149.8 | 141.8 |  | 82.8 | 109.6 |
|  | ε | -144 |  | -98.5 | -140.2 |  | -163.4 | -150.6 |  | -166 | -118 |
|  | ζ | -127.1 |  | -79.4 | -170.1 |  | 149.2 | 160.6 |  | -139 | 142 |
|  | χ | -174.8 |  | 29 | -169.9 |  | -127.7 | -145.7 |  | 177.5 | -107.8 |
| T12 | α | -105.9 |  | -74.2 | -80 |  | -138.1 | -165.5 |  | -107.1 | 76 |
|  | β | -103.2 |  | -162.8 | -157.3 |  | -145.6 | -125 |  | -135.1 | 108.5 |
|  | γ | 62.8 |  | 51.7 | 55.6 |  | 68.4 | 68.2 |  | 72.7 | -17.7 |
|  | δ | 145.9 |  | 110.7 | 150.6 |  | 141.3 | 144.3 |  | 89.3 | 92.2 |
|  | ε | 173.1 |  | -150.5 | -173.2 |  | -163.4 | -134 |  | 56.2 | 34.5 |
|  | ζ | -103.9 |  | -123 | -75.9 |  | 132.8 | 81.6 |  | -100.7 | 158.8 |
|  | χ | -120.7 |  | -155.8 | -132.5 |  | -126.4 | -112 |  | 14.4 | 28.4 |
| G13 | α | -73.9 |  | -16.9 | -82.1 |  | -55.4 | 143.9 |  | -81.4 | -94.3 |
|  | β | -169.7 |  | 161.8 | 165.7 |  | 151.1 | -136.6 |  | -95.9 | -125.2 |
|  | γ | 62 |  | 23.9 | 59.7 |  | -53 | 52.4 |  | -56.4 | 73.7 |
|  | δ | 133.7 |  | 140.7 | 104.7 |  | 143.8 | 123.5 |  | 160.3 | 145.7 |
|  | ε | 149.9 |  | 177.8 | -179.3 |  | -162.2 | -170.1 |  | -173.3 | -164.7 |
|  | ζ | -89.6 |  | -107 | -105.3 |  | -105.3 | -108.5 |  | -103.2 | -130.3 |
|  | χ | -113.1 |  | -110.1 | -139.4 |  | -124.4 | -122.4 |  | -134.7 | -124.9 |
| G14 | α | -70.3 |  | -63.9 | -58.2 |  | -75.2 | -63.4 |  | -123.8 | 161.2 |
|  | β | -144 |  | -175.8 | -178.3 |  | 177.4 | -179 |  | -165.5 | -145.4 |
|  | γ | 51.9 |  | 50.6 | 45.7 |  | 55.1 | 47.9 |  | 96 | 161.1 |
|  | δ | 142.4 |  | 141.2 | 132.4 |  | 138.3 | 133.9 |  | 132.2 | 103.8 |
|  | ε | 160.8 |  | -176.5 | 171.9 |  | -165 | -174.6 |  | 173 | -178.9 |
|  | ζ | -109.4 |  | -108.8 | -107.9 |  | -126.9 | -111.1 |  | -104.6 | -86.4 |
|  | χ | -109 |  | -112.6 | -115.5 |  | -108.7 | -112.5 |  | -120.3 | -150.9 |
| G15 | α | 173 |  | -64.9 | -56.5 |  | -62.5 | -61.1 |  | -51.2 | -65.6 |
|  | β | -160 |  | -170.7 | -163.1 |  | 171.2 | -172 |  | -151.7 | -155.4 |
|  | γ | 165.5 |  | 45 | 44.4 |  | 52.5 | 42.9 |  | 33 | 39 |
|  | δ | 131.7 |  | 141.7 | 144.2 |  | 135.2 | 142.6 |  | 156.2 | 148.9 |
|  | ε | -179.5 |  | -179.7 | -178.3 |  | -168 | -167.6 |  | 175.1 | -173.9 |
|  | ζ | -72.7 |  | -96.9 | -80.9 |  | -96.6 | -97.6 |  | -89.7 | -103.7 |
|  | χ | -140.4 |  | -109.5 | -116.6 |  | -118 | -111.5 |  | -107.2 | -113.4 |
| A16 | α | -89 |  | -51.3 | -67.1 |  | -58.7 | -67.9 |  | -62 | -52.3 |
|  | β | -169.2 |  | 165 | 170.9 |  | 171.2 | 168.9 |  | -167.6 | 177.7 |
|  | γ | 61.3 |  | 48.4 | 49.6 |  | 52.1 | 61.5 |  | 48.7 | 49.2 |
|  | δ | 135.1 |  | 139 | 142.8 |  | 138.4 | 139.6 |  | 150 | 154.6 |
|  | ε | -179 |  | -176.2 | -176.6 |  | -174.7 | -173.8 |  | 174.5 | 171.3 |
|  | ζ | -81.1 |  | -98.9 | -97.8 |  | -98 | -90.8 |  | -99.4 | -91.3 |
|  | χ | -97 |  | -93.5 | -96.9 |  | -94.9 | -92.6 |  | -79.3 | -85.1 |
| G17 | α | -61.8 |  | -63.6 | -70 |  | -62.7 | -61.4 |  | -47.9 | -62.4 |
|  | β | 159.6 |  | 174 | 164 |  | 161.1 | 171.6 |  | 172.3 | -178.3 |
|  | γ | 68.7 |  | 60.6 | 67.5 |  | 64.7 | 57.5 |  | 49.5 | 58.1 |
|  | δ | 131.9 |  | 154.2 | 144.7 |  | 143.2 | 140.9 |  | 150.8 | 141.5 |
|  | ε | -177.8 |  | -95.8 | -106.4 |  | -128.8 | -93.4 |  | -101.7 | -132.5 |
|  | ζ | -125.2 |  | 160.4 | 157.8 |  | -171.9 | 148.5 |  | 173.6 | 169.7 |
|  | χ | -109.5 |  | -84 | -104.1 |  | -102.7 | -91.3 |  | -92.5 | -92.4 |
| G18 | α | -50.5 |  | -68.9 | -30.7 |  | -57.3 | -95.8 |  | -86.8 | -40.6 |
|  | β | 148.6 |  | 135.4 | 112.1 |  | 143.1 | 161.4 |  | 149 | 127.9 |
|  | γ | 67.8 |  | 60 | 43.5 |  | 44 | 55.2 |  | 50.3 | 40.4 |
|  | δ | 123.7 |  | 151.7 | 135.5 |  | 138.3 | 151.4 |  | 142.2 | 147 |
|  | ε | -140.7 |  | -113.3 | -94.6 |  | -70.2 | -68.2 |  | -71.7 | -91.4 |
|  | ζ | 171 |  | 57.1 | 94.8 |  | 96.2 | 104.3 |  | 95.6 | 118.4 |
|  | χ | -127.3 |  | -83.4 | -94.6 |  | -120.1 | -126 |  | -111.9 | -112.2 |
| A19 | α | 64.9 |  | -142.9 | 175 |  | 60.9 | 153.3 |  | 45.1 | 92.7 |
|  | β | -133.8 |  | -138.4 | -175 |  | 135.8 | -128.3 |  | 140.4 | -176.4 |
|  | γ | 175.6 |  | 54.5 | 65.4 |  | 165.9 | 50.3 |  | -179.3 | 122 |
|  | δ | 140.4 |  | 105.8 | 95.5 |  | 136.4 | 147.8 |  | 140.1 | 106.8 |
|  | ε | -159.9 |  | -95.6 | -106.6 |  | -89 | -156.3 |  | -101 | -78.7 |
|  | ζ | 164.5 |  | -66.4 | -59.2 |  | -64.1 | -86.9 |  | -61.9 | -82.5 |
|  | χ | -48.5 |  | -75.3 | -77.1 |  | 104.1 | -178 |  | 120.2 | 116.5 |
| G20 | α | 97.8 |  | -86.7 | -89.9 |  | -84.1 | -103.7 |  | -90.7 | -77.7 |
|  | β | -93 |  | 78.3 | 83.4 |  | 78.1 | 85.1 |  | 74.3 | 54.3 |
|  | γ | 177.5 |  | -177.5 | 179 |  | -179.2 | -177.7 |  | -177.4 | -172.7 |
|  | δ | 132.1 |  | 115.1 | 125.3 |  | 120 | 121 |  | 128.4 | 131.8 |
|  | ε | -117.3 |  | -154.3 | -151.8 |  | -145.2 | -151.2 |  | -144.1 | -152.8 |
|  | ζ | -73.4 |  | -88.3 | -91.8 |  | -87.7 | -102.1 |  | -98.7 | -89 |
|  | χ | -113.8 |  | -97.7 | -91.1 |  | -96.5 | -85.5 |  | -88.4 | -87.6 |
| G21 | α | -67.2 |  | 177.3 | 166.4 |  | 174.4 | 170.3 |  | 164.1 | -117.5 |
|  | β | 165.3 |  | 179.7 | -177.8 |  | -179 | -173.7 |  | 175.8 | -148.4 |
|  | γ | -57.5 |  | 38.1 | 44.3 |  | 40.3 | 45.8 |  | 60.6 | -34.9 |
|  | δ | 129.6 |  | 100.9 | 93.5 |  | 94.8 | 94.5 |  | 94.5 | 136.4 |
|  | ε | -148.5 |  | -133 | -140.9 |  | -139.1 | -139.3 |  | -118.4 | -139.1 |
|  | ζ | -82.4 |  | -75.8 | -72 |  | -70.6 | -74.1 |  | -79.5 | -62.6 |
|  | χ | 176.8 |  | 164.7 | 166.9 |  | 166.4 | 166.3 |  | 158.3 | 176.6 |
| G22 | α | -83.2 |  | -66.9 | -72.2 |  | -71 | -71.5 |  | -59.4 | -69.5 |
|  | β | -155.8 |  | -167.2 | -163.9 |  | -153 | -163.7 |  | -178 | -162.9 |
|  | γ | 46.7 |  | 44.8 | 52.9 |  | 46.9 | 53.8 |  | 39.1 | 31.8 |
|  | δ | 145.3 |  | 147.7 | 145.1 |  | 150.5 | 153 |  | 149.6 | 154.7 |
|  | χ | -101.4 |  | -107.2 | -107.3 |  | -107.5 | -113.4 |  | -114.9 | -107.2 |

**Table S2.** Structural stability of the five c-KIT G4 systems studied by molecular dynamics simulations. Each of the 250 ns MD runs was performed in triplicate, for a total of 3750 ns simulation time.

| **System** | **250 ns MD** | **cKIT native** | | **cKIT brom.(3qxr)** | | **cKIT NMR (2o3m)** |
| --- | --- | --- | --- | --- | --- | --- |
| **chain** |  | **nat-A** | **nat-B** | **Br-A** | **Br-B** | **model 1** |
| **composition** |  | **22mer+3K^+^** | **22mer+2K^+^** | **22mer+4K^+^+2Mg^2+^** | **22mer+4K^+^** | **22mer+2K^+^** |
| RMSD G4 vs start | run #**1** | 2.76 Å | 3.28 Å | 2.48 Å | 2.57 Å | 1.85 Å |
| RMSD G4 vs start | run #**2** | 3.30 Å | 3.78 Å | 2.30 Å | 3.02 Å | 1.86 Å |
| RMSD G4 vs start | run #**3** | 3.00 Å | 3.06 Å | 2.60 Å | 3.01 Å | 1.88 Å |
| RMSD G4 vs avg | run #**1** | 1.29 Å | 1.41 Å | 1.45 Å | 1.24 Å | 1.25 Å |
| RMSD G4 vs avg | run #**2** | 1.44 Å | 1.37 Å | 1.32 Å | 1.64 Å | 1.19 Å |
| RMSD G4 vs avg | run #**3** | 1.51 Å | 1.41 Å | 1.55 Å | 1.21 Å | 1.12 Å |
